# Post-transcriptional screen of cancer amplified genes identifies *ERBB2*/*Her2* signaling as AU-rich mRNA stability-promoting pathway

**DOI:** 10.1038/s41389-021-00351-w

**Published:** 2021-09-17

**Authors:** Jenan Al-Matouq, Latifa Al-Haj, Maher Al-Saif, Khalid S. A. Khabar

**Affiliations:** 1 Molecular BioMedicine Program, King Faisal Specialist Hospital and Research Centre, Riyadh, 11211 Saudi Arabia; 2 Present Address: Mohammed Al-Mana College for Medical Science, Dammam, Saudi Arabia

**Keywords:** Post-translational modifications, Cancer genetics

## Abstract

Amplification of specific cancer genes leads to their over-expression contributing to tumor growth, spread, and drug resistance. Little is known about the ability of these amplified oncogenes to augment the expression of cancer genes through post-transcriptional control. The AU-rich elements (ARE)-mediated mRNA decay is compromised for many key cancer genes leading to their increased abundance and effects. Here, we performed a post-transcriptional screen for frequently amplified cancer genes demonstrating that ERBB2/Her2 overexpression was able to augment the post-transcriptional effects. The ERBB1/2 inhibitor, lapatinib, led to the reversal of the aberrant ARE-mediated process in ERBB2-amplified breast cancer cells. The intersection of overexpressed genes associated with ERBB2 amplification in TCGA datasets with ARE database (ARED) identified ERBB2-associated gene cluster. Many of these genes were over-expressed in the ERBB2-positive SKBR3 cells compared to MCF10A normal-like cells, and were under-expressed due to ERBB2 siRNA treatment. Lapatinib accelerated the ARE-mRNA decay for several ERBB2-regulated genes. The ERBB2 inhibitor decreased both the abundance and stability of the phosphorylated inactive form of the mRNA decay-promoting protein, tristetraprolin (ZFP36/TTP). The ERBB2 siRNA was also able to reduce the phosphorylated ZFP36/TTP form. In contrast, ectopic expression of ERBB2 in MCF10A or HEK293 cells led to increased abundance of the phosphorylated ZFP36/TTP. The effect of ERBB2 on TTP phosphorylation appeared to be mediated via the MAPK-MK2 pathway. Screening for the impact of other amplified cancer genes in HEK293 cells also demonstrated that EGFR, AKT2, CCND1, CCNE1, SKP2, and FGFR3 caused both increased abundance of phosphorylated ZFP36/TTP and ARE-post-transcriptional reporter activity. Thus, specific amplified oncogenes dysregulate post-transcriptional ARE-mediated effects, and targeting the ARE-mediated pathway itself may provide alternative therapeutic approaches.

## Introduction

Amplification of certain oncogenes can increase the expression of specific cancer mediators that participate in tumor progression and maintenance [1]. Although many mechanistic details for several amplified driver genes have been explored, little is known about their effects on post-transcriptional regulation of gene expression. Post-transcriptional processes can be compromised in cancer, including defective AU-rich element (ARE)-mediated mRNA decay leading to over-expression of the cancer-promoting genes [2]. Thus, it is important to explore the deleterious effects of amplified oncogenes on post-transcriptional events. One of the key amplified oncogenes found in particularly breast, stomach, esophageal, and uterine cancers is ERBB2 (also called *HER2*/*Neu*), which we found here to cause AU-rich mRNA stabilization in breast cancer cells. *ERBB2* amplification in breast cancer constitutes 20−30% of all breast cancer cases and is associated with enhanced tumor aggressiveness and reduced patient survival [3]. Therefore, investigating new interactions in this pathway is important for further understanding this disease and, ultimately, its management and therapy.

*ERBB2* is a member of the epidermal growth factor receptor family comprising the transmembrane tyrosine kinase receptors (EGFR or ERBB1) and ERBB2 to ERBB4. Upon their activation, they form homodimerization or heterodimerization of the receptor, and subsequent phosphorylation of the receptor domain c-terminal tyrosine residues occurs [4]. These events lead to the activation of several intracellular signaling pathways that include the PI3K/AKT, PLCγ/protein kinase-C (PKC), Ras/MAPK/ERK, and JAK/STAT pathways [5, 6]. In turn, these signaling pathways activate cancer gene products that promote hallmarks of cancer, including increased proliferation, invasion, angiogenesis, metastasis, and resistance to therapy [7–9].

The RAS-MAPK kinase pathway is one of the signaling cascades activated by ERBB2 and is suggested to be in concert with the PI3K/AKT pathway in ERBB2-positive breast cancer [10, 11]. One target for MAPK pathway-stimulated phosphorylation is tristetraprolin (TTP, also called ZFP36); it is an RNA-binding protein that generally promotes the decay of AU-rich mRNAs [12, 13]. When phosphorylated, it becomes inactive, leading to increased mRNA stability and enhanced translation [14, 15]. Because ZFP36/TTP is deficient in cancer—both as abundance and activity-consequent increased mRNA stability and translation of AU-rich mRNA of many cancer genes are found, contributing to hallmarks of cancer [2, 16]. Here, we explore the effect of amplified cancer genes on ARE- mRNA pathways and found that ERBB2 is a significant mediator of perturbed activity in post-transcriptional control of gene expression.

## Results

### Screening of amplified cancer genes for post-transcriptional effects

**Fig. 1:**
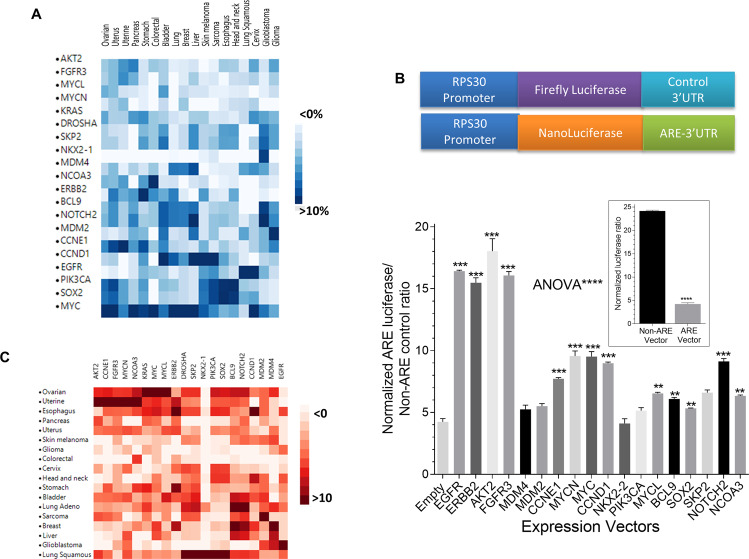
A screen for post-transcriptional regulation of genes containing AREs elements. **A** A gene-centric heatmap representing copy number abundance (a minimum of 10% frequency in at least one single type of cancer). Color legend represents % frequency. **B** HEK293 cells were co-transfected with Super Nanoluciferase reporter vectors containing non-ARE control or RPS30-nLuc-ARE reporter together with of control Firefly luciferase vector, for 18 h. Cells were re-seeded into 96-well microplates and were transfected with empty vector control or *ERBB2*-HA vector for 18 h. Cells were lysed, and luciferase activity was quantitated as the ratio of Nanoluciferase/Firefly luciferase intensity. The screen is at least from two independent experiments with Mean ± SEM of triplicate readings. ANOVA with Dunnett’s multiple comparisons was used to compare the effects of each of the indicated vectors and the empty vector on ARE-reporter readings. **p < 0.01, ***p < 0.001, ****p < 0.0001. **C** Cancer-centric heatmap representing clustering of cancer type according to copy number variations.

In order to focus on the most frequent amplified oncogenes for studying post-transcriptional effects, we analyzed the frequency of amplified cancer genes with a minimum 10% occurrence in at least one cancer type of the eighteen TCGA databases. Figure 1A shows a heatmap of 20 gene-centric clusters as a result of this analysis. Subsequently, we assessed the effect of forced expression of the most frequently amplified cancer genes on the AU-rich element (ARE)-mediated pathway using our optimized reporter model of selective post-transcriptional control [17, 18] in HEK293 cells. When overexpressed, several cancer genes caused an increase in the ARE-mediated reporter activity compared with an empty vector (Fig. 1B). The most potent effect (nearly 15-fold, *p* < 0.0001) was due to the expression of EGFR and ERBB2 genes, its downstream effector AKT2, and FGFR3 (Fig. 1B). Other cancer amplified genes that also significantly increase ARE reporter activity are MYC (a previously known gene to exert post-transcriptional effect [19]), MYCN, CCND1, CCNE1, SKP2, and NOTCH2 (Fig. 1B). The cancer-centric cluster of the amplified oncogenes indicated that ERBB2/EGFR/AKT is predominately found in breast cancer (Fig. 1C). We then chose to focus this study on ERBB2, an important molecule in the pathogenesis and targeted therapy of ERBB2/HER2 + breast cancer.

### Systematic analysis of ERBB2-associated ARE-coding genes in breast cancer

**Fig. 2:**
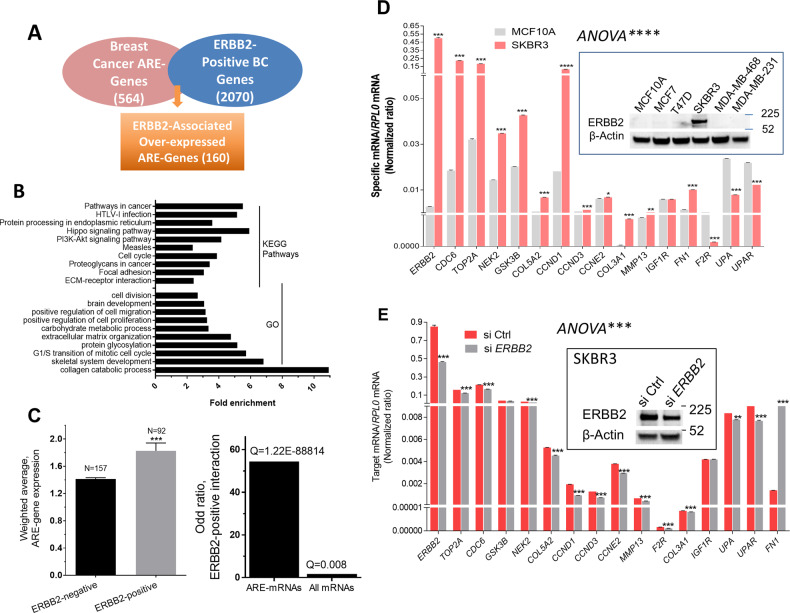
Systematic analysis of ERBB2-associated ARE-coding genes in breast cancer. **A** The ERBB2-amplification associated expressed genes (≥1.7 fold, *Q* value <0.00005) were intersected with the AU-rich element database (ARED-Plus). **B** Functional enrichment of the ERBB2-associated ARE-coding genes. The most significant results with false discovery rate (FDR) *P* < 0.05 are shown. **C** Left panel: the mean of mRNA abundance for 160 ARE-coding genes (Fig. 1A data) was compared between TCGA ERBB2-negative and ERBB2-positive patients’ tissue samples. Data are Mean ± SEM. ***p < 0.001 using Student’s t-test with Welch’s correction. Right panel: the interaction between the ARE-mRNA cluster or all mRNAs with ERBB2-positive gene expression data is represented by an odd ratio using Fisher’s exact test (Oncomine software). **D** Total RNA from the ERBB2-positive SKBR3 cell line and MCF10A normal-like ERBB2-negative cell lines were extracted and subjected to RT-QPCR using specific primers to the indicated genes. Inset shows an immunoblot of two for the ERBB2 protein among different types of breast cancer cell lines. **E** The SKBR3 cells were transfected with 200 nM control siRNA or ERBB2 siRNA for 24 h, followed by RNA extraction and RT-QPCR as indicated. Inset is a representative immunoblot of three, for the ERBB2 protein. The data in D and E are normalized to the housekeeping gene, RPL0. Data are Mean ± SEM (triplicate) from one representative experiment of at least two. ANOVA test demonstrates overall statistical significance. The student’s t-test was used to compare expression data for each gene.

We intersected an updated list of 564 over-expressed breast cancer ARE-mRNAs based on our previous analysis [16] with a list of genes that selectively over-expressed in ERBB2-positive invasive ductal breast cancer tissues (TCGA database). The intersection result is 160 ERBB2-associated ARE-mRNA cluster (Fig. 2A and Supplementary Table 1). Functional analysis revealed enrichment of specific categories mostly related to two hallmarks of cancer: cell cycle and invasion/metastasis (Fig. 2B). Invasion and metastasis are represented by several functional groups, including cell migration, extracellular matrix organization, and focal adhesion (Fig. 2B). The PI3-AKT kinase pathway, which is known to be regulated by ERBB2 [20], is also over-represented. Analysis of TCGA patients’ tissue data of ERBB2-amplified invasive ductal breast cancer showed significant upregulation of the ERBB2-associated ARE-mRNA cluster compared to ERBB2-negative samples (Fig. 2C, left panel). Moreover, substantial enrichment of ARE-mRNAs was observed, when compared to overall mRNAs in the ERBB2-positive group (Fig. 2C, right panel).

For further analysis, we selected a group of genes to compare the mRNA abundance of these genes in the ERBB2-negative MCF10A normal-like cells and SKBR3 cell line, a well-established cellular model of ERBB2-amplified cancer. Many of these genes, except a few, were over-expressed in the ERBB2-amplified SKBR3 cells by at least 2.0-fold (Fig. 2D). In particular, highly expressed genes, including *CCND1*, *CDC6*, *COL3A1*, *COL5A2*, *FN1*, *MMP13*, and *TOP2A* with more than five-fold abundance, were observed. As a result of *ERBB2* knockdown by siRNA interference, the mRNA abundance, each of *CCND1*, *CCND3, CCNE2, CDC6*, *COL5A2*, *FN1*, *MMP13, TOP2A*, *UPA*, *F2R*, and *UPAR* was decreased in the ERBB2-positive SKBR3 breast cancer cells (Fig. 2E).

### The ERBB1/ERBB2 inhibitor, lapatinib, reduces ARE-mRNA abundance and stability

**Fig. 3:**
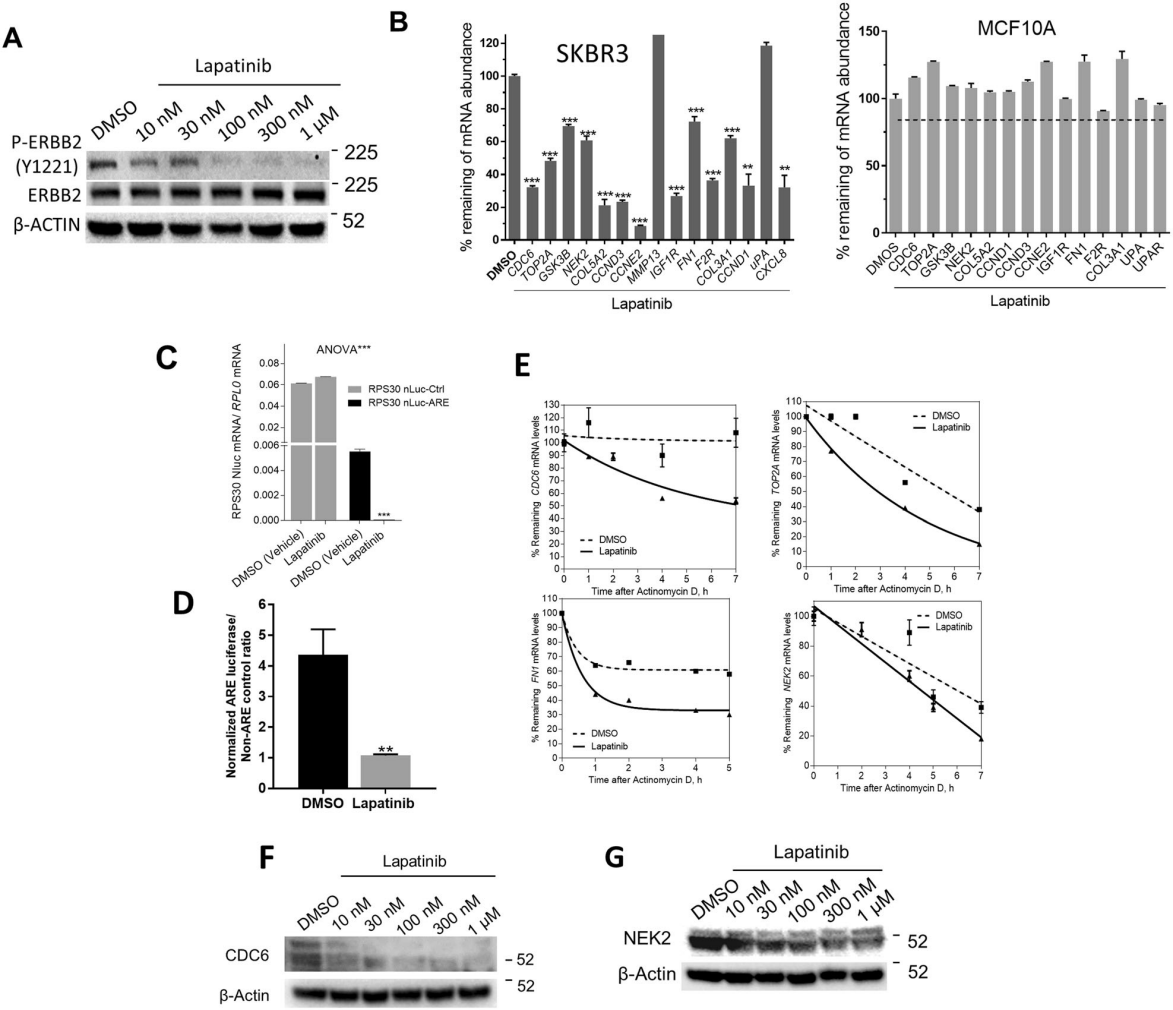
The ERBB1/ERBB2 inhibitor, lapatinib, normalizes aberrant ARE-mediated post-transcriptional control. **A** SKBR3 cells were treated with increasing doses of lapatinib for 24 h, and lysed for immunoblotting with anti-phospho ERBB2 protein and β-actin as a loading control. WB is one representative from three blots. **B** RT-QPCR expression analysis for the ERBB2-regulated ARE-mRNAs in SKBR3 cells (left panel) or MCF-10A cells (*right panel*) treated with DMSO or Lapatinib (300 nM) for overnight. Data are normalized to the housekeeping gene, RPL0. The normalized values were used to calculate % remaining relative to DMSO control. Statistical analysis was performed using two-tailed Student’s *t*-test for each gene reduced in expression in comparison to DMSO data. Data are Mean ± SEM (triplicate of one experiment of three). **P < 0.001, ***P < 0.0001. **C** RT-QPCR for Nanoluciferase mRNA abundance or Nanoluciferase protein activity (**D**) in SKBR3 cells transfected with nLuc-ARE or control nLuc-non-ARE expression plasmid for overnight, and then treated with DMSO (control) or lapatinib for 6 h or 24 h, respectively. Data are presented as the mean ± SEM (*n* = 2 experiments). ANOVA is used to compare overall significance while post hoc sidak’s test compares the DMSO and lapatinib data. ***p < 0.0001. **p < 0.005, Student’s test. **E** The mRNA half-life determination in response to lapatinib. The SKBR3 cells were treated with DMSO or lapatinib (300 nM) overnight, then actinomycin D (AcD) (5 μg/ml) was added for the indicated time points. Total RNA was extracted, and cDNA samples were subjected to RT-QPCR using specific primers to the indicated genes. One-phase exponential decay curves for relative mRNA half-life measurements in the two treated groups are shown. Data are normalized to *RPL0* transcript abundance, and the normalized values were used to calculate the mRNA half-life as a percentage to time 0. Data are presented as the mean ± SEM (*n* = 2 experiments). **F**, **G** Immunoblot analysis for CDC6 and NEK2 protein abundance in SKBR3 cells treated as in (**A**). Representative blot each is from at least two experiments is shown.

We tested the effect of the tyrosine kinase inhibitor lapatinib on the abundance of the ERBB2-associated ARE-mRNA group. First, we confirmed the dose-dependent effect of lapatinib on ERBB2 phosphorylation in the SKBR3 cell line (Fig. 3A). In most of our experiments, we chose log_1.5_ (330 ng/ml) concentration, which is within the therapeutic levels of lapatinib [21]. We found that the mRNA abundances of most ERBB2-associated ARE-coding genes (except uPA and MMP13) were reduced by the ERBB1/ERBB2 inhibitor and ranged from 30 to 90% reduction in gene expression levels (Fig. 3B, left panel). In contrast, lapatinib had no or minimal reducing effects on the ARE-mRNA expression in the ERBB2-negative MCF10A (Fig. 3B, right panel).

Treatment of SKBR3 cells with the ERBB1/2 tyrosine kinase inhibitor, lapatinib, significantly decreased the ARE-reporter mRNA abundance (*P* < 0.0001; Fig. 3C) as well as the reporter protein activity (Fig. 3D). Furthermore, the mRNA half-life change due to lapatinib was examined for several genes. The drug reduced the mRNA half-life of the tested genes (*CDC6*, *TOP2A*, *FN1*, and *NEK2*, Fig. 3E). The reduction of *CDC6* and NEK2 mRNA levels by lapatinib was also reflected at the protein level, and in a dose-response manner (Fig. 3F, G). Together, these results indicated that the lapatinib imparts a post-transcriptional control effect mediated by AREs.

### The ERBB2 pathway leads to ZFP36/TTP phosphorylation

**Fig. 4:**
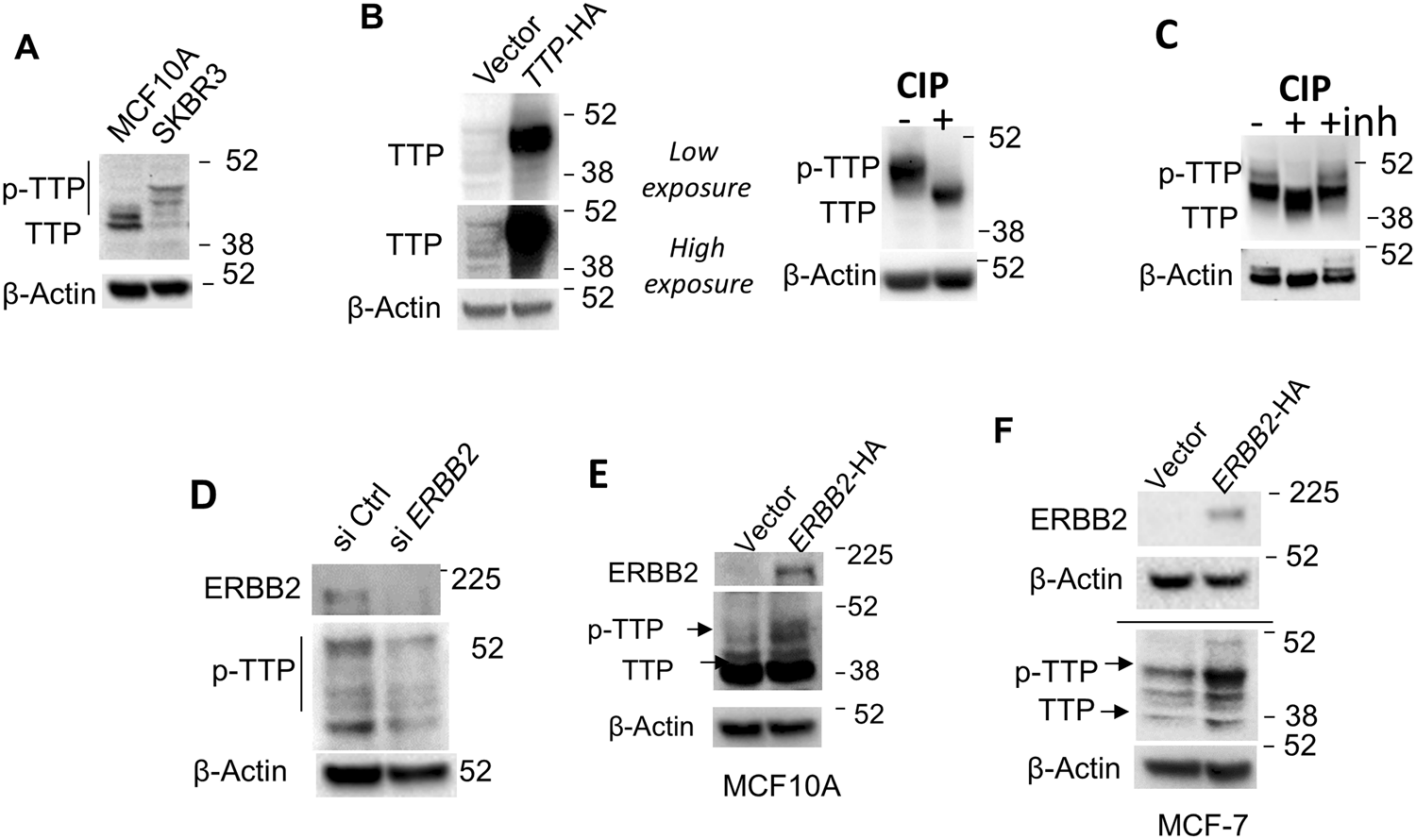
ERBB2 signaling-mediated phosphorylation of ZFP36/TTP. **A** immunoblot analysis for ZFP36/TTP phosphorylated protein abundance in MCF10A and SKBR3 using specific primers to ZFP36/TTP and β-Actin protein as a loading control. **B** SKBR3 cells were transfected with an empty vector or ZFP36/TTP expression vector. Left panel: proteins were extracted, and immunoblotting was performed using a ZFP36/TTP antibody. Right panel: protein lysates were first treated with the phosphatase, CIP, or CIP phosStop **C** before immunoblotting with anti-ZFP36/TTP. The WB shows both phosphorylated and unphosphorylated bands. WB was performed with low exposure to avoid high background and overexposed signal of the transfected TTP (lower panel). D) SKBR3 cells were transfected with 200 nM *ERBB2* siRNA or control siRNA for overnight, and *ERBB2* and ZFP36/TTP protein abundance were assessed by immunoblotting. A representative blot from at least two experiments is shown. Immunoblot analysis of ERBB2 and ZFP36/TTP protein phosphorylation abundance in MCF10A (**E**) or MCF-7 (**F**) transfected with ERBB2 expression vector or empty vector. Representative blots are from at least three experiments.

Because ZFP36/TTP is a key RNA-binding protein whose phosphorylation results in attenuation of its RNA decay activity [22], we have examined the modulation of ZFP36/TTP by ERBB2 pathway. To investigate this further, we used a custom-designed antibody that recognizes ZFP36/TTP protein. We developed a special western blot with run conditions to increase the sensitivity of detecting both unphosphorylated (lower molecular weight of nearly ~40 kDa) and phosphorylated ZFP36/TTP, which ran at appreciably higher molecular weight [23]. Most of the band in SKBR3 cell line belongs to the higher phosphorylated ZFP36/TTP when compared to the normal-like ERBB2-negative MCF-10A (Fig. 4A). Similarly, when SKBR3 was transfected with ZFP36/TTP expression plasmid, the over-expressed band was of the higher molecular weight (Fig.4B, left panel). This is further confirmed when the phosphatase, CIP, was utilized to treat the protein extracts with, the CIP-treated band shifted to the low molecular weight band that represents unphosphorylated ZFP36/TTP (Fig. 4B, right panel). Additional phosphatase inhibitor control reversed the shift (Fig. 4C). Knockdown of ERBB2 in SKBR3 resulted in a reduced abundance of phosphorylated ZFP36/TTP forms (Fig. 4D). In contrast to ERBB2 inhibition, over-expression of ERBB2 in the normal-like ERBB2-negative MCF-10A and MCF-7 tumor line led to increased abundance of phosphorylated ZFP3/ZFP36/TTP protein (Fig. 4E, F).

### The ERBB1/ERBB2 inhibitor, lapatinib, reduces ZFP36/TTP phosphorylation and protein stability

**Fig. 5:**
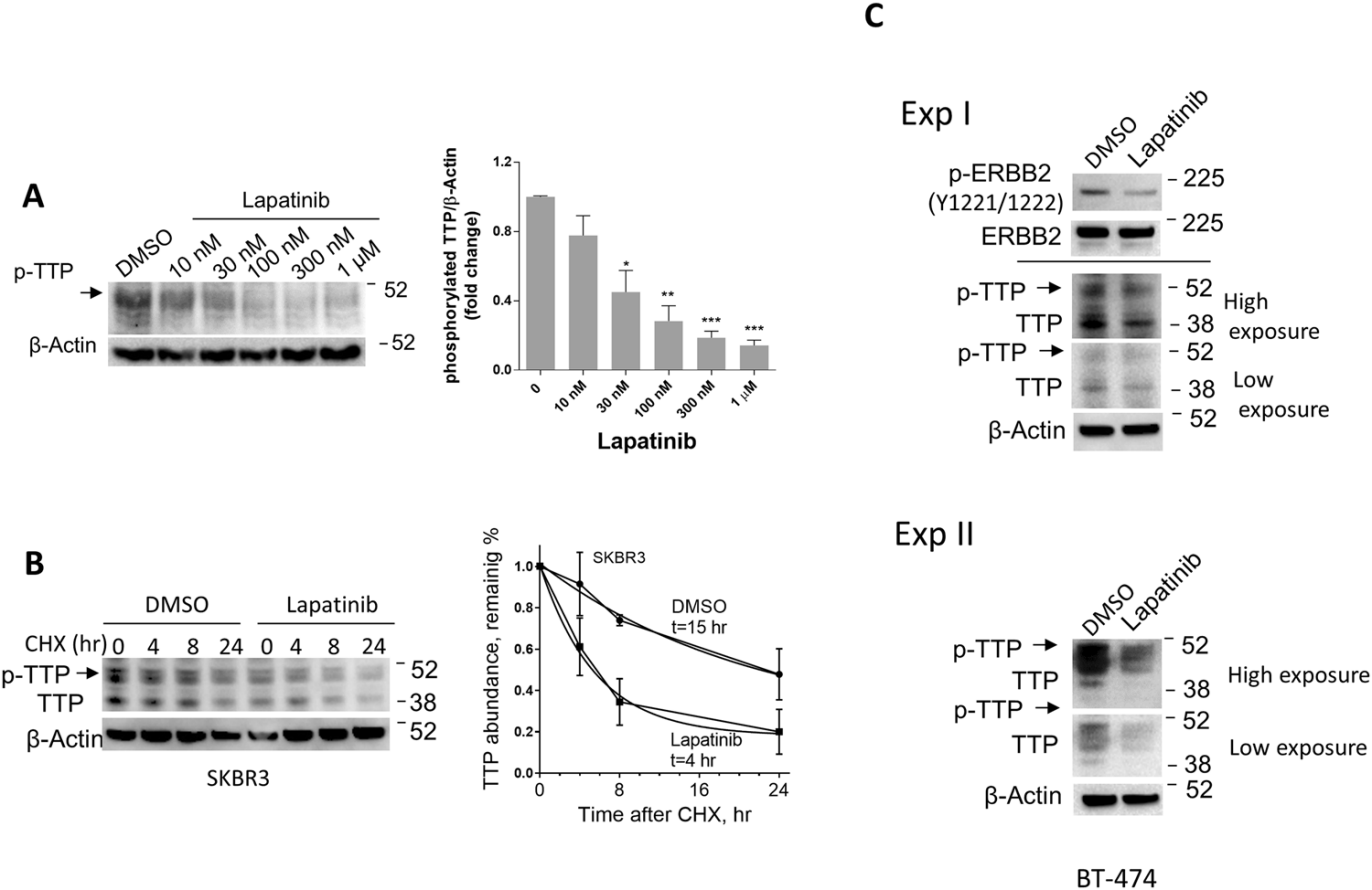
Effect of ERBB2 pharmacological inhibitor on ZFP36/TTP phosphorylation. **A** Dose-dependent activity of lapatinib on the abundance of phosphorylated ZFP36/TTP in the ERBB2-positive SKBR3 cells. The relative amounts of the proteins from three immunoblots were quantified using Image J software (*left panel*). Fold changes (Mean ± SEM, *n* = 3) of phosphorylated ZFP36/TTP abundance normalized to β-Actin using Image J program (right panel). **B** Effect of ERBB2 inhibition on ZFP36/TTP protein stability. SKBR3 cells were treated with lapatinib (300 nM) for 16 h, followed by 10 µg/ml of cycloheximide (CHX) treatment for the indicated durations (left panel). Protein lysates were subjected to immunoblotting with anti-TTP. Right panel: quantification of ZFP36/TTP abundance, normalized to GAPDH, was determined using ImageJ software and the protein half-life was assessed using the Graph Prism. Data are mean +/− SEM of two experiments. **C** Effect of lapatinib on the abundance of ZFP36/TTP protein in the ERBB2-positive cells, BT-474. WB is a representative of two blots from independent experiments.

Lapatinib was able to reduce the abundance of phosphorylated ZFP36/TTP in the ERBB2-positive SKBR3 cells and in a dose-dependent manner (Fig. 5A). The considerable loss of ZFP36/TTP protein due to ERBB2 inhibition is likely due to the degradation of TTP protein as it becomes unphosphorylated. It has been thoroughly demonstrated that the phosphorylated form of TTP is stable and considerably degraded upon loss of phosphorylation [24, 25]. Indeed, when we performed CHX-chase experiment in SKBR3 cells, lapatinib substantially reduced the protein half-life of ZFP36/TTP (Fig. 5B). The effect of lapatinib on the abundance of phosphorylated ZFP36/TTP was also demonstrated with another ERBB2-positive breast cancer cell line, BT-474 (Fig. 5C).

### Effect of ERBB2 inhibition on signaling pathways leading to ZFP36/TTP phosphorylation

**Fig. 6:**
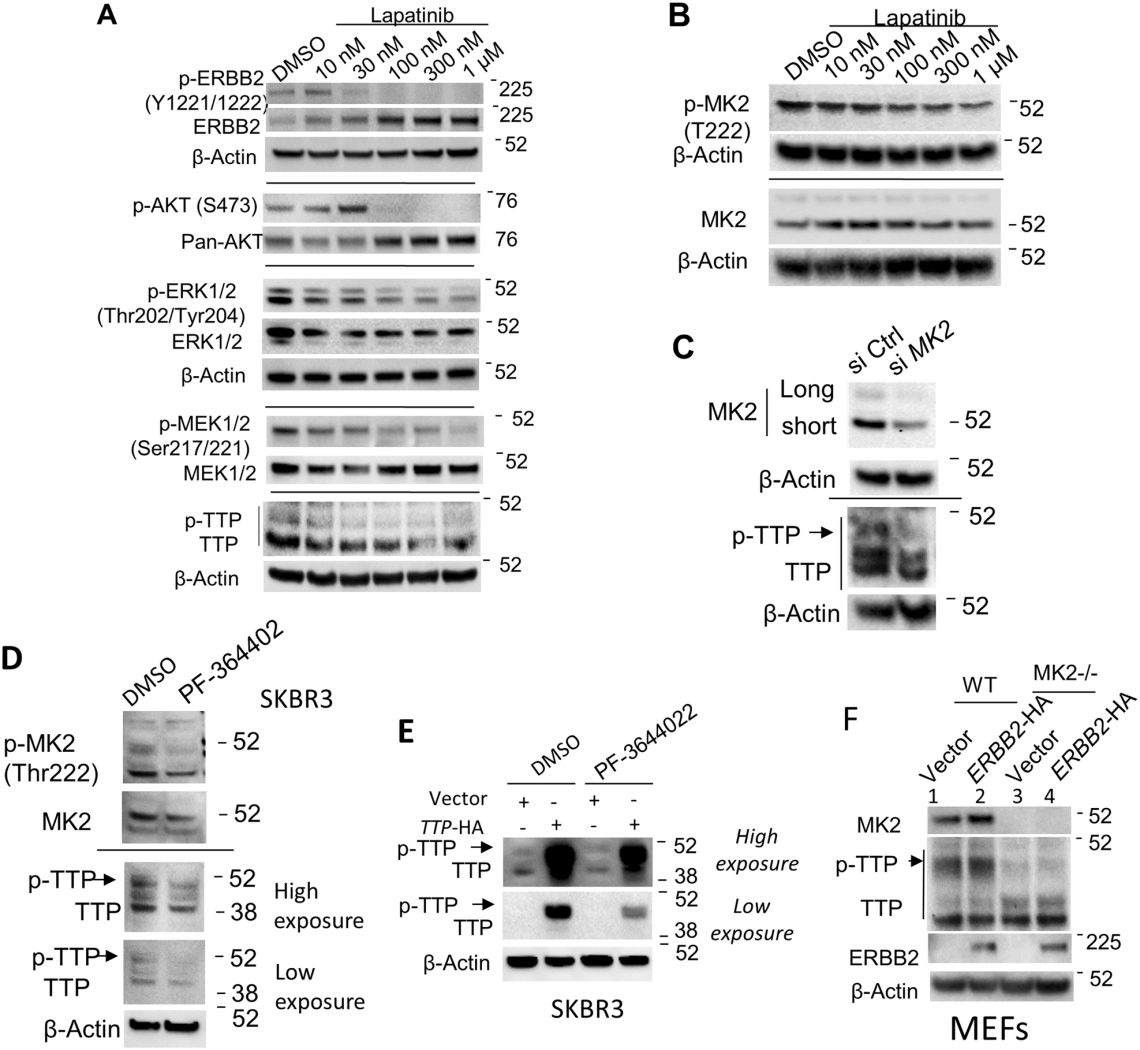
ERBB2/MK2 signaling pathway mediated protein phosphorylation of ZFP36/TTP. **A** Immunoblot analysis of ERBB2, AKT, MEK1/2, and ERK1/2 signaling inhibition after treatment with increasing doses of lapatinib for 24 h. Status of ERBB2 (phospho-tyrosine 1221/1222 and total), AKT (phospho-serine 473 and total pan AKT), ERK1/2 (Phospho-threonine 202/phospho-tyrosine 204 and total), MEK1/2 (Ser217/221), and ZFP36/TTP are shown. A blot from at least two experiments is shown. **B** Immunoblot analysis for the inhibition of MK2 activation after treatment with lapatinib. SKBR3 cells were treated with increasing doses of lapatinib, as indicated for 24 h. Phospho-threonine MK2 (T222) and total MK2 protein abundance are shown. A representative blot from two experiments is shown. **C** Immunoblot analysis for ZFP36/TTP phosphorylation in *MAPKAPK-2* silenced SKBR3 cells. SKBR3 cells transfected with 100 nM of si Ctrl or si *MAPKAPK-2* for 24 h were tested for ZFP36/TTP protein abundance. Total MK2, phosphorylated ZFP36/TTP, and β-actin protein abundances are shown. A representative blot from three experiments is shown. Effect of the MK2 inhibitor PF-3644022 (1 µM, 4 h) on the abundance of phosphorylated ZFP36/TTP in ERBB2-expressing SKBR3 (**D**) or SKBR3 transfected with ZFP36 expression plasmid (**E**). Western blots are from two independent experiments. **F** Immunoblot analysis of total and phosphorylated ZFP36/TTP, and MK2 in *MK2*-knockout MEF cells in the absence or presence of overnight transfected ERBB2.

Taken together, the above experiments showed that ERBB2 signaling activity leads to an observed increase in the abundance of the higher molecular weight (phosphorylated) ZFP36/TTP, suggesting the presence of downstream effector kinases in the ERBB2 pathway that can cause ZFP36/TTP phosphorylation. These kinases are, namely, the MAPK-activated protein kinase 2 (MK2), extracellular signal-regulated kinase (ERK1/2), and AKT kinases [22, 24, 26–28]. Thus, we have looked at the status of these kinases during lapatinib treatment. Lapatinib appears to downregulate all the phosphorylated kinases known to affect ZFP36/TTP phosphorylation, with doses higher than 30 nM were effective (Fig. 6A, B). These effects paralleled the reduction of the phosphorylated ZFP36/TTP abundance (Fig. 6A, lower panel).

**Role of MAPK-MK2 pathway in ERBB2-mediated TTP phosphorylation and stability**

Specifically, the silencing of the MAP kinase MK2 in SKBR3 cells led to a reduction in the abundance of phosphorylated ZFP36/TTP, indicating this pathway’s involvement in ERBB2-amplified cancer cells (Fig. 6C). The pharmacological MK2 phosphorylation inhibitor, PF-3644022, reduced the abundance of phosphorylated ZFP36/TTP in the ERBB2-amplified SKBR3 cells (Fig. 6D). To signify the weak TTP bands, we over-express ZFP36/TTP in SKBR3 cells and demonstrated similarly that the MK2 inhibitor decreases the abundance of phosphorylated ZFP36/TTP protein (Fig. 6E). Utilizing MEFs that are knockout of *MK2* gene, there was a strong requirement for MK2 in the appearance of phosphorylated ZFP36/TTP protein (lanes 1 and 3, Fig. 6F). When ERBB2 was introduced in MEFs, only in MK2 background, there was an increased abundance of phosphorylated ZFP36/TTP, while loss of phosphorylated ZFP36/TTP was maintained in the *MK2*-knockout MEFs (Fig. 6F). These results together may suggest the involvement of ERBB2-MK2-ZFP36/TTP in ERBB2/*Her2*-amplified breast cancer.

### Amplified genes in ERBB2/EGFR/AKT pathway and TTP/ZFP36 phosphorylation

**Fig. 7:**
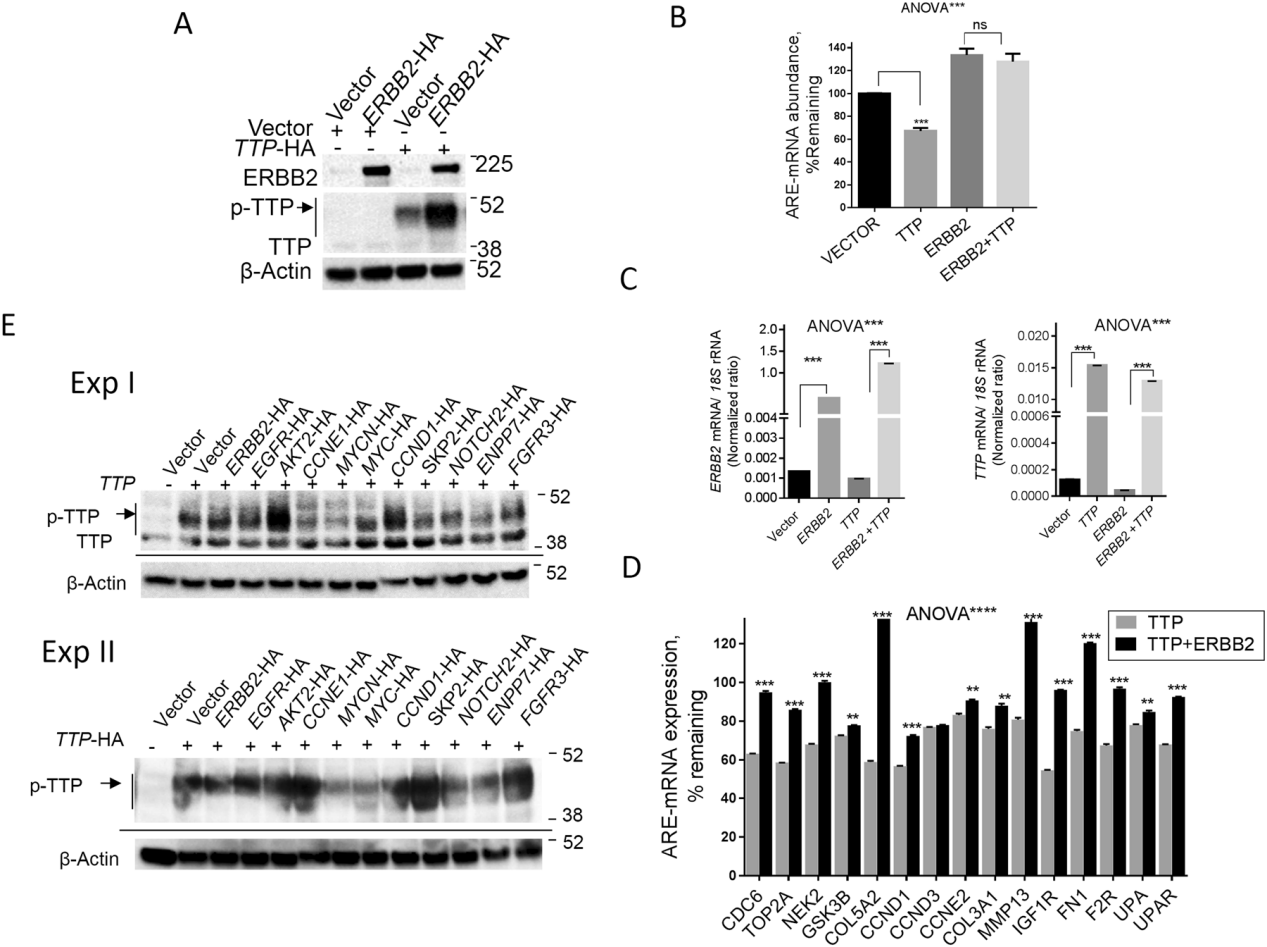
Effects of amplified cancer genes on ZFP36/TTP phosphorylation. **A** HEK293 cells were co-transfected with *ERBB2*-HA along with *ZFP36/TTP*-HA or vector control (0.5 µg each) for 24 h. Proteins were extracted, and immunoblotting was performed using antibodies to ERBB2, ZFP36/TTP, and β-actin. A representative blot from three experiments is shown. **B** Total RNA samples were extracted and subjected to RT-QPCR using specific primers to the ERBB2-regulated ARE-mRNA s (as in Fig. 2D, E). Data are Mean ± SEM of average 18SRNA normalized expression of ARE-mRNA abundance remaining from vector control. **C**, **D** RT-QPCR using specific primers to the indicated genes RT-QPCR using specific primers to the indicated genes. Data in **B** to **D** are Mean ± SEM from an experiment with triplicate reactions of two independent experiments. Two-way ANOVA with Tukey’s multiple tests between the control and treatment test as indicated were used. ***p < 0.001. **E** Immunoblot analysis for ZFP36/TTP protein in HEK293 cells over-expressing cancer-amplified genes. HEK293 cells were co-transfected with cancer amplified genes and *ZFP36/TTP*-HA or vector control (0.5 µg each) for 24 h. The results of the two experiments are shown.

When both ERBB2 and ZFP36/TTP were co-expressed in HEK293 cell line that lacks these proteins, a significant increase in the abundance of the phosphorylated ZFP36/TTP was seen (Fig. 7A), confirming the experiments above. Moreover, we have tested in these experiments the effect of ZFP36/TTP on the ARE-mRNAs in the presence or absence of ERBB2. Although, ZFP36/TTP reduced the expression of ARE-mRNAs only modestly and by an average of 30% (Fig. 7B), the inclusion of forced ERBB2 expression largely abolished the TTP effect corresponding to the increased abundance of the phosphorylated protein (Fig. 7B). The mRNA abundance of forced TTP and ERBB2 in the transfected cells is shown as controls (Fig. 7C). The behavior of individual ARE-mRNAs in response to ZFP36/TTP, in the presence or absence ERBB2 expression, demonstrates that the modest reduction of ARE-mRNA by TTP mostly reversed due to ERBB2 introduction (Fig. 7D).

Using the HEK293 co-expression model as in vivo phosphorylation of ZFP36/TTP, we also investigated the effect of the amplified oncogenes that were positives in increasing AU-rich element-mediated reporter activity (Fig. 1B). We found that the following amplified oncogenes, EGFR, AKT2, CCND1, CCNE1, SKP2, and FGFR3, can increase the abundance of phosphorylated ZFP36/TTP (Fig. 7D). Together with the effects of these amplified oncogenes on the reporter data, the ERBB2/EGFR/AKT pathway appears to be a major player in ARE-mediated post-transcriptional regulation in relevant cancers.

## Discussion

AREs play a major role in the post-transcriptional control of gene expression, which has been increasingly shown to be defective in cancer. For example, ARE-mRNAs have been identified to be over-expressed and over-represented in cancer [16] and many participate in all hallmarks of cancer [2, 29]. Thus, studying the effects of certain oncogene amplifications that frequently occur in cancer on the post-transcriptional control of ARE-mRNA is important. In this study, we found that several frequently amplified oncogenes led to the upregulation of gene expression at the post-transcriptional level, as evaluated by specific ARE-mediated reporter activity. Several amplified oncogenes that impart post-transcriptional effect are MYC, MYCN, CCND1, CCNE1, NOTCH2, SKP2, ENPP7, and FGFR3. Among these that previously studied for the post-transcriptional effects is MYC. This oncogene exerts an indirect effect on ARE-mediated events by inhibiting the transcription of ZFP36/TTP, which promotes ARE-mRNA decay [19]. MYC amplification is found across many types of cancers with an estimated overall frequency of 14% [30]. Like MYC being a transcriptional factor with high homology [31, 32], MYCN may also inhibit ZFP36/TTP transcription. Among those amplified oncogenes with the strongest effects on the ARE-linked reporter activity are ERBB2, EGFR, and AKT2, which are components of PI3K/AKT and RAS/MEK/ERK pathways. These amplified oncogenes are predominately observed in ERBB2-positive tumors. Thus, we focused on ERBB2 amplification effects on ARE-mediated post-transcriptional control.

The ARE-mRNA cluster of ERBB2 (160 genes) found here is significantly over-represented when compared to the overall ERBB2-positive expressed genes and comprises many genes that are important in several cancer processes. A notable functional enrichment is related to the invasiveness of the cells. Over-expression of ERBB2 in cancer is strongly linked to the cancer invasive capacity and patient’s survival [33, 34]. Because ZFP36/TTP is deficient in abundance and activity-in cancer cells, including invasive ductal breast cancer, over-expression of ERBB2-positive genes is further augmented by prolonged mRNA stability and increased translation. We have previously identified several invasion-promoting ARE-mRNAs that are post-transcriptionally aberrant due to ZFP36/TTP deficiency, including PLAU, PLAUR, MMP13, and CXCR4 [35, 36], in which many are ERBB2-regulated genes. Several ERBB2-associated ARE-mRNAs identified here are linked to the invasive processes of focal adhesion and extracellular matrix modeling such as FN1, COL5A2, COL3A1, CCND1, and CCND3 [5, 37]. These genes are also mediated by the PI3K-AKT signaling, a pathway that is highly regulated by ERBB1 (EGFR)/ERBB2 [38, 39]. Other sets of ERBB2-associated ARE-mRNAs are related to the proliferative activity of the cells. We and others have shown that ZFP36/TTP act as S/G2 and G2/M tumor suppressor, which become compromised in invasive breast cancer, and many cell cycle genes are upregulated in a post-transcriptional manner [16, 40]. Some of these genes appeared here as ERBB2-regulated and included CDC6, NEK2, and TOP2A.

The ERBB1/ERBB2 drug, lapatinib, reduced the expression of the majority of the tested ARE-mRNAs-except uPA and MMP13. The lapatinib-inhibited gene group is mostly parallel to those of the ERBB2 siRNA-inhibited group in ERBB2-amplified SKBR3 cells. Moreover, the ARE-mRNA coding group in ERBB-2 positive SKBR3 is highly abundant compared to the ERBB2-negative MCF10A. It is not surprising finding in our results that uPA-uPAR system is not consistently involved in ERBB2-amplified cancer since an earlier report showed that ERBB2 overexpression did not affect uPA expression [41]. Lapatinib was able to normalize the prolonged mRNA half-life in ERBB2-amplified cancer cells for several selected ARE-mRNAs, which confirms the ARE reporter assay data.

Our results demonstrate that the ERBB2 pathway leads to ZFP36/TTP phosphorylation using different cell lines. We found that ZFP36/TTP was phosphorylated mainly in breast cancer cell lines, including ERBB2-amplified SKBR3, and demonstrated that lapatinib—also ERBB2 siRNA-reduced the abundance of the phosphorylated ZFP36/TTP in a dose-dependent manner. These results corresponded well with prior work and our recent report [42], demonstrating that TTP protein is stable when phosphorylated, particularly in tumor cells and during inflammatory response [26, 27, 43, 44].

Indeed, several experimentally validated are mRNA targets for ZFP36/TTP including CDC6, TOP2A, NEK2, MMP13, CCND1, and IL-8 [16, 19, 28, 35, 45]. The phosphorylated ZFP36/TTP is inactive in promoting mRNA decay by preventing deadenylation through sequestering by 14-3-3 protein [12, 46, 47]. Besides ERBB2, we found that other certain amplified oncogenes, EGFR, AKT2, CCND1, CCNE1, SKP2, and FGFR3, can increase the abundance of phosphorylated ZFP36/TTP. These would need further to be explored in future studies.

The Ras/MAPK/ERK, MAPK-MK2, and PI3 kinase/AKT pathways are known to cause ZFP36/TTP phosphorylation and subsequent ARE-mRNA stabilization [22, 24, 26–28, 44, 48, 49]. These pathways are also maintained by ERBB2 signaling [10, 11]. Thus, our studies demonstrate that these same pathways being reduced upon ERBB2 inhibition, are associated with the reduced abundance of phosphorylated ZFP36/TTP. In particular, MK2 is a master regulator for ZFP36/TTP phosphorylation in inflammation and cancer (reviewed in: [49, 50]). Our results show that ERBB2 signaling appears to cause ZFP36/TTP phosphorylation in a manner that is, at least partially, dependent on MK2 phosphorylation. Thus, a potential ERBB2-MK2-ZFP36/TTP phosphorylation axis is operative in ERBB2-amplified cancers, which can be targeted by inhibitors of the MAPK-MK2 pathway. Our data demonstrate the importance of some sets of amplified oncogenes in the post-transcriptional regulation of gene expression and pinpointed a new factor in ERBB2-mediated signaling, which is the increased abundance of ZFP36/TTP phosphorylation.

## Materials and methods

### Datasets and bioinformatics

The Catalog of somatic mutations in cancer (COSMIC) http://cancer.sanger.ac.uk/cosmic and the cBioPortal for Cancer genomics http://www.cbioportal.org), for data mining were used. We analyzed the frequency of amplified cancer genes with a minimum 10% occurrence in at least one single cancer type from the eighteen TCGA databases. Heatmaps were generated using centroid hierarchal clustering algorithm in JMP statistical package (SAS Institute, US). Using the cBioPortal, we interrogated the invasive breast cancer TCGA datasets for *ERBB2* gene amplification and analyzed the associated enrichment of gene expression (RNAseq data and microarray expression intensities level 2 data). The *ERBB2*-amplification associated expressed genes (≥ 1.7 fold, *Q* value <0.00005) were intersected with the AU-rich element database (ARED-Plus) using LIST search at https://brp.kfshrc.edu.sa/ared to derive *ERBB2*-amplified associated ARE-mRNA coding genes. Functional enrichment analysis was performed using DfreqAVID bioinformatics https://david.ncifcrf.gov/.

### Cell lines and reagents

The human breast ERBB2-expressing adenocarcinoma cell lines SKBR3 and BT-474, ERBB2-negative MCF-7 cell line, breast normal-like cell line MCF10A, and the Human embryonic kidney cells (HEK293) cell lines were obtained from American Type Culture Collection (ATCC; Rockville, MD). Cells were obtained and stored in June 2016 and were tested for authentication before publication on (Jan 2021) using polymorphic short tandem repeat (STR) examination. This analysis utilized multiplex PCR (Promega PowerPlex^®^ 1.2 System), which amplify eight loci (vWA, TH01, TPOX, CSF1PO, D13S317, D16S539, D7S820, and D5S818) plus Amelogenin for gender determination. Mycoplasma screening was routinely performed using a PCR kit (Boca Scientific, USA). MK2-knockout mouse embryo fibroblasts(MEF) were kindly provided by Dr. M. Gaestel (Germany). HEK293, MCF-7, and MEFs were cultured in Dulbecco’s modified Eagle’s medium (DMEM; ThermoFisher) supplemented with 10% fetal bovine serum, 100U/ml penicillin, 100 μg/ml streptomycin, and 2 mM L-Glutamine. SKRB3 cells were cultured in McCoy’s 5a Medium Modified (ThermoFisher) supplemented with 10% fetal bovine serum, 100U/ml penicillin, 100 μg/ml streptomycin, and 2 mM L-Glutamine. MCF10A cells were cultured in a 1:1 mixture of Ham’s F12 and DMEM supplemented with 10% fetal bovine serum, 0.01 mg/ml bovine insulin, 100 ng/ml cholera toxin, 20 ng/ml human epidermal growth factor, and 500 ng/ml hydrocortisone (Sigma, St Louis, MO). Lapatinib ditosylate (GW572016/Tykerb, Selleckchem, USA) was obtained from Selleckchem and prepared with dimethyl sulfoxide (DMSO). PF-3644022 (MK2 inhibitor) was purchased from Tocris (Tocris Bioscience, Minneapolis, MN).

### RNA, reverse transcription, and real-time PCR

Total RNA samples were extracted using Trizol reagent (ThermoFisher) according to the manufacturer’s instructions. Reverse transcription reaction was performed using 5 µg total RNA with Superscript II first-strand synthesis system (ThermoFisher). RT-QPCR was performed using Taman gene expression assays (Applied Biosystems, CA, USA) and 5X Taqman master mix (Al-Bio, KSA) according to the manufacturer’s instructions. As an internal control, we used a VIC-labeled *RPL0* mRNA probe (Applied Biosystems). Samples were amplified in triplicate in a CFX96 cycler (Bio-Rad); quantification of relative expression was performed using the ΔΔ*C*_t_ method.

### mRNA half-life determination

Actinomycin D (ActD, 5 µg/ml; Sigma) was added to the cells for 1, 2, 4, and 7 h prior to extraction of total RNA. The mRNA half-life determinations were calculated using the one-phase exponential decay method using GraphPad Prism software (GraphPad Software, San Diego, CA). This method has the best fit for mRNA data that tend to decay at a certain rate over a period of time and then reach a plateau; all fits were >*R*^*2*^ = 0.9.

### RNA interference, plasmids, and transfection

siRNAs were custom synthesized by Metabion (Martinsried, Bavaria, Germany). The control siRNA is (Sense: 5′-GAUAUGUCAACUCAG UACUtt-3′ and antisense: 5′-AGUACUGAGUUG ACAUAUCtt-3′ and ERBB2 siRNA is (Sense: 5′-GAGUCCCAACCAUGUCAAAtt-3′ and antisense: 5′-UUUGACAUGGUUGGGACUCtt-3′). Lipofectamine 2000 reagent (ThermoFisher) was used for transfection with 50 nM of the siRNA. The indicated plasmids of the amplified genes, including ERBB2-HA, were purchased from Sino Biologicals (China). The HA-ZFP36/TTP plasmid was described previously [16]. For the plasmid transfection experiment, cells were transfected with 0.5 µg expression vector per million cells using lipofectamine 2000 according to the manufacturer’s instructions.

### Antibodies and western blotting

Custom rabbit anti-ZFP36/TTP polyclonal antibody was custom synthesized against a short peptide in the C terminus by GenScript (USA). We used an NP-40-based lysis buffer optimized for detecting both phosphorylated and total ZFP36/TTP protein [42]. Briefly, cells were washed with ice-cold Phosphate buffer Saline (PBS) and centrifuged at 2000 rpm for 5 min at 4 °C. Next, an equal volume of NP-40 lysis buffer (50 mM Tris-HCl pH 8.0, 150 mM NaCl, 1% IGEPAL CA-630, plus EDTA-free protease inhibitor cocktail (Sigma) and phosphatase inhibitor (PhosStop, Sigma) were added to the cell pellet and incubated on ice for 10 min. Samples were sonicated and centrifuged for 10 min at maximum speed at 4 °C. Purified protein samples were used to detect ZFP36/TTP protein abundance. RIPA buffer or NP-40 lysis buffer was used to detect other proteins as well. Calf intestinal phosphates (CIP, Sigma) treatment of protein lysates was performed as previously performed and according to the standard protocol [24, 26, 27, 42]. In addition, we used PhosStop as an additional control.

Antibodies used in this study are rabbit anti-Phospho threonine-MAPKAPK-2 (Thr222) (9A7) mAb; rabbit anti-Phospho tyrosine-HER2/ErbB2 (Tyr1221/1222) mAb; and rabbit anti-MAPKAPK-2 mAb and were obtained from Cell Signaling Technology (Beverly, MA, USA). Rabbit anti-CDC6 [clone DCS-180] antibody was obtained from Sigma (St Louis, MO, USA). Mouse anti-HER2/ErbB2 mAb [3B5] and mouse anti-beta actin antibody [mAbcam 8226] were obtained from (Abcam Inc., MA, USA). Rat anti-HA high-affinity mAb [clone 3F10] was obtained from Roche Applied Science (USA).

### Protein stability assay

SKBR3 cells were seeded overnight and then treated with CHX (Sigma-Aldrich) at a concentration of 30 μg/mL for 0, 4, 8, and 16 h. Cells were collected, and the protein abundance was determined by immunoblotting, and the β-actin normalized quantification was performed using ImageJ software. The protein half-life was determined using regression analysis with Graph-Pad software.

### 3′UTR and ARE nano-luciferase reporter assay and nano-luciferase RT-QPCR

Cells were seeded into three 10 cm plates for overnight, then transfected with 6 µg of the following constructs: (non-ARE-nanoluciferase, ARE-nanoluciferase, and ARE-3′UTR-nano-luciferase reporters) using lipofectamine 2000 [51]. Twenty-four hours after transfection, cells in each 10 cm plate were trypsinized and seeded into four wells of six-well plate and incubated overnight. Next, cells were treated with (DMSO or Lapatinib) for 6 h (RNA) or 16 h (nanoluciferase activity). Total RNA was extracted, and cDNA was used to detect luciferase mRNA abundance with Nanoluciferase specific Taqman primers and probes (forward: 5′-CTCCATCTTCG CGGTAGCT-3′; reverse: 5′- GAGGACTTGGTCCAGGTTGTA-3′; and probe: 5′- 6-FAM-CCGCCGTTC AGTCGCCGT-BHQ-1-3′. For the luciferase activity, cells were lysed and assayed with the dual Nano-Glo luciferase kit (Promega) according to the manufacturer’s instructions and measured on a plate luminometer. Data were presented as Mean ± SEM of normalized Nano luciferase intensity/Firefly intensity.

### Statistical analysis

All data are present as Mean ± SEM of at least three experiments as stated otherwise in the Figure legends. Statistical significance was calculated using a two-tailed Student’s t-test when comparing two columns –Welch’s correction for variance was applied in case of different variances- or ANOVA when comparing three columns of data or two-way ANOVA when compared column data with more than one independent factor. Post hoc multiple comparison tests were used with ANOVA. Statistical and graphical data was performed with GraphPad Prism software (San Diego, CA)].

## Supplementary information

Supplementary Table S1
